# Bioprocessing to Preserve and Improve Microalgae Nutritional and Functional Potential: Novel Insight and Perspectives

**DOI:** 10.3390/foods12050983

**Published:** 2023-02-26

**Authors:** Michela Verni, Chiara Demarinis, Carlo Giuseppe Rizzello, Erica Pontonio

**Affiliations:** 1 Department of Soil, Plant and Food Science, University of Bari Aldo Moro, Giovanni Amendola 165/A, 70126 Bari, Italy; 2 Department of Environmental Biology, “Sapienza” University of Rome, Piazzale Aldo Moro 5, 00185 Rome, Italy

**Keywords:** bioprocessing, microalgae, nutritional improver, food functionality

## Abstract

Microalgae are aquatic unicellular microorganisms and, although various species are approved for human consumption, *Arthrospira* and *Chlorella* are the most widespread. Several nutritional and functional properties have been bestowed to microalgae principal micro- and macro-nutrients, with antioxidant, immunomodulatory and anticancer being the most common. The many references to their potential as a food of the future is mainly ascribed to the high protein and essential amino acid content, but they are also a source of pigments, lipids, sterols, polysaccharides, vitamins, and phenolic compounds with positive effects on human health. Nevertheless, microalgae use is often hindered by unpleasant color and flavor and several strategies have been sought to minimize such challenges. This review provides an overview of the strategies so far proposed and the main nutritional and functional characteristic of microalgae and the foods made thereof. Processing treatments have been used to enrich microalgae-derived substrates in compounds with antioxidant, antimicrobial, and anti-hypertensive properties. Extraction, microencapsulation, enzymatic treatments, and fermentation are the most common, each with their own pros and cons. Yet, for microalgae to be the food of the future, more effort should be put into finding the right pre-treatments that can allow the use of the whole biomass and be cost-effective while bringing about features that go beyond the mere increase of proteins.

## 1. Introduction

The challenge of feeding the growing world population and the necessity to provide a nutritionally balanced diet while reducing greenhouse gas emissions, as well as a transition to a diet higher in plant- rather than animal-derived proteins, are the driving force toward changes in consumers’ dietary pattern and the current evolution of the food industry. Although the demand for meat and dairy products is bound to grow by 2050, under current average production practices, this approach is ultimately not sustainable due to the related increased green-house gas emissions [1].

Plant-based products are nutritionally valuable sources of protein, but they require land and water, both of which will become limited over time. Furthermore, plant proteins are converted to meat proteins rather inefficiently since ~6 kg of plant proteins are required to produce 1 kg of meat proteins [2]. In this framework, single cell proteins, the bulk of dried cells (biomass) produced by algae, yeast, bacteria, and fungi, represent a viable alternative to classical protein sources [3]. Microalgae are a heterogeneous group of eukaryotic organisms such as chlorophylls (green algae), bacillaryiophytes (diatoms), dinophytes (dinoflagellates), euglenophytes, prymnesiophytes (cocolitophorides), and prokaryotes [4] present in both marine and freshwater environments [5], with some species also distributed in humid soils [6]. Microalgae cultivation provides high-value biomass, while the existing diversity of microalgae species allows the production of different types of products. Hence, microalgae are biomass with applications in the pharmaceutical, cosmetic, and food industries, and as micro-factories in the form of cell or cell-factories/bio-factories [7].

Currently, *Spirulina platensis* (*Arthrospira* spp., world annual production 5000 tons of dry weight) and *Chlorella vulgaris* (world annual production 2000 tons of dry weight) are the most widely cultivated species [8] because they are classified as Generally Recognized As Safe (GRAS) according to the Food and Drug Administration (FDA), as well as validated by the World Health Organization as “superfoods” [9] and sold as healthy food worldwide [10]. Nevertheless, other species, e.g., *Nannochloropsis* spp. and *Haematococcus pluvialis* [11,12], are also produced and studied, but their exploitation depends on food safety regulations, market demand, commercial factors, and specific preparation [13].

Microalgae can boost several nutritional features, considering either the basic nutrition provided through proteins, lipids, and carbohydrates, and additional health benefits [14] thanks to high-value bioactive primary and secondary metabolites [15,16].

Interest in research in the use of microalgae as either food or ingredient has a long history; indeed, circa 5664 (Scopus, last access on 20 January 2023) documents, with the words “microalgae” and “food” in either the title, abstract or key words, have been published since 1969, respectively, with an exponential trend in the past ten years. Circa 9637 documents were patented from 2013 with more than 1200 documents/year since 2017 (Google Patent, last access on 14 February 2023).

Nevertheless, problems related to nutrient accessibility and availability, metabolite extraction and purification, and sensory quality in formulated food products have driven research projects toward the optimization of bioprocesses for the production, pre-treatment, and incorporation of microalgae in food. The current review’s aim is to provide a comprehensive discussion of the bioprocesses optimized for microalgal production and utilization in the food industry given the main drawbacks related to its use for human consumption. A detailed description of the nutritional composition of micro-algae can be obtained in some complementary literature [16].

## 2. Regulatory Aspects

Although the consumption of some microalgal and cyanobacterial species as foodstuff or food supplements has been common practice for centuries, being traditionally used as human food in many regions and countries [17], their use as food and/or ingredients is regulated by several European Union [18] and other countries (East Asia, Australia, and the USA), corresponding to national regulations [19].

In Europe, to be commercialized, every food or ingredient must be authorized by the European Food Safety Authority (EFSA). This step is crucial given the ability of some algae to produce toxins harmful to humans [4]. For this reason, foods containing all or part of the algal biomass must comply with the Novel Food Regulation (EU) 2015/2283. This regulation establishes a watershed date: 15 May 1997. Only foods or ingredients that were not on the market before this date must comply with the Regulation on Novel Foods and Novel Food ingredients (Regulation (EC) No. 258/97). *Chlorella* and *Arthrospira*, for example, have been on the market since before 1997, but to sell a new component extracted from them, it is necessary to apply for authorization from EFSA, which must assess its safety. Plancton Marino Veta la Palma^®^ (Fitoplancton Marino S.L., Cádiz, Spain), which is the dried biomass of *Tetraselmis chuii*, for example, has been recently authorized by EFSA to be marketed as a Novel Food in accordance with Article 3(1) of Regulation (EC) No 258/97 [20]. Similarly, *Aurantiochytrium limacinum* and *Euglena gracilis* consumption was recently approved by EFSA [4].

In the USA, food safety is ensured by the FDA, which applies two main regulations to microalgae and food, (i) the Federal, Drug and Cosmetic Act, and (ii) the Dietary Supplement Health and Education Act. *Arthrospira* sp. and *Chlorella* sp. have been granted GRAS status by the FDA [4].

Asian countries have highlighted the benefits of algae for centuries, and due to their rich cultural history, consumers are more aware of the potential of microalgae compared to Western countries. Several active microalgae cultivation companies located in China, Japan, Taiwan, Thailand, and India are growing and many more projects are in the research and development phase [21]. In China, seaweed, microalgae, and cyanobacteria are used as health foods which are regulated under the Food Safety Law, amended in 2015 and enforced by the National Medical Products Administration (formerly the China Food and Drug Administration) [21]. In Japan, research activities and mass cultivation of Spirulina for use as a foodstuff began in the 1970s and its food safety is governed by the Minister of Health, Labor and Welfare as part of the Department of Food Safety, under the Pharmaceutical and Food Safety Bureau, and foods with health claims are either classified as Foods with Nutrient Function Claims or Foods for Specified Health Uses [21].

## 3. Microalgae Bioprocessing

### 3.1. Production

**Figure 1:**
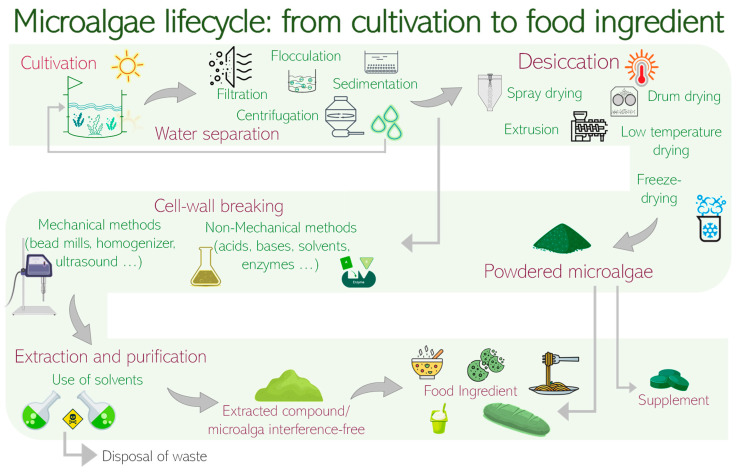
The microalgae production process. All phases leading to use as food ingredients are included.

The production of algal biomasses involves several steps, each one affecting the quality of the final product (Figure 1).

The whole process can be summarized in three phases: cultivation, harvesting, and extraction. This last step is important if the algal biomass is not used in its entirety, yet bioactive compounds need to be extracted from it. Cultivation can take place in open-air ponds or in closed bioreactors. To choose between these two methods, several variables must be considered: the nature of the microalgae, the availability of nutrients, the climate, and the final uses of the biomass [22]. Microalgae cultivation also requires specific environmental conditions including temperature ranges, light intensities, mixing conditions, nutrient composition, and gas exchange. They can be cultured using different metabolic pathways (photoautotrophic, heterotrophic, and mixotrophic) and by using different cultivation systems, commonly classified as open and closed systems (for reviews see Zuccaro et al. [23] and Daneshvar et al., 2021 [24]).

To separate the microalgae from the growth medium, the harvesting phase can be carried out using different techniques: filtration, ultrafiltration, centrifugation, and sedimentation, combined with flocculation, or fluctuation-flocculation [25]. The harvesting method will also influence the quality of the final product [26]. For low quality products, sedimentation followed by flocculation is mainly used; for high quality products, such as those used in feeding and food, continuous fractionation centrifuges are preferred. The harvesting method also affects the final biomass density: for example, sedimentation may be used to obtain a dilute biomass [25,26].

Because of their high-water content and, therefore, high perishability, drying processes are necessary to preserve algae biomass, although often associated with loss of quality compared to the fresh product. The drying phase can be carried out through drum drying, freeze-drying, spray-drying, and sun-drying [26]. During this phase, carotenes and fatty acids can be oxidized [27]. To avoid this problem, Cyanotech Corp. has developed a protocol in which a closed system with a flow of nitrogen and carbon dioxide is used to keep the oxygen concentration low, allowing a drying process for algal biomass in less than six seconds [27].

Desmorieux and Hernandez [28] studied various methods of drying Spirulina and compared the amount of protein before and after processing. Freeze-drying showed the lowest protein loss, more than 90% of the initial proteins remaining in the dry product. All other methods (convective and oven drying, infrared, and spray drying) resulted in 10–25% protein losses. The effect of desiccation methods on *Arthospira platensis* were also assessed by Verni et al. [29]. The authors compared the protein profile, through chromatographic and electrophoretic techniques, of wet, dried at low temperature, and lyophilized Spirulina, observing remarkable lower contents of peptides and free amino acids in dried biomass, compared to fresh biomass. Indeed, before drying, excess water is drained through filtration. Even though drying at low temperature does not impact the nutritional properties as higher temperatures could do, a large part of the soluble compounds, among which are amino acids and peptides, is lost anyway. On the contrary, increases of peptides and amino acids (up to 180 mg g^−1^ combined) were observed in lyophilized compared to wet Spirulina, probably due to the combination of the freeze-storage and freeze-drying processes which led to the lysis or damage of the cells [29].

### 3.2. Drawbacks for Human Consumption

Several nutritional and functional properties have been bestowed to microalgae principal micro- and macro-nutrients, with antioxidant, immunomodulatory, and anticancer being the most common (Table 1).

The many references to their potential as a food of the future is mainly ascribed to the high protein and essential amino acid content, but they are also a source of pigments, lipids, sterols, polysaccharides, vitamins, and phenolic compounds with positive effects on human health [30,31,32]). Nevertheless, despite such positive effects, their commercial uptake is still limited, mainly due to the green color and fishy smell and taste [20]. Culinary traditions also play a key role: in many Asian countries the addition of microalgae in food products is very common; on the contrary, in Europe microalgae are not traditional ingredients, and their use is considered as a noticeable change in food production [33]. To overcome these issues, several solutions could be applied. Extracting the targeted biomolecules, instead of using the entire algal biomass for food, would help in achieving the desired feature, while the food keeps its original color [20]. The fishy taste could be covered using exotic-flavored spices, or discoloration techniques can be applied. Encapsulation has also been suggested as a useful tool to mask the unpleasant aroma of microalgae and for the suppression of several other limitations (e.g., high sensitivity to processing conditions, short shelf-life, fast-release of flavor during storage, limited uptake and bioavailability, lack of compatibility and uniformity with the food matrix, or degradability through the gastrointestinal tract passage), since it enables the protection of a wide range of compounds by their entrapment into a protective matrix. The structure of an encapsulation system depends upon the arrangement of the core substance and deposition process of the coating material, which can be broadly divided into reservoir or matrix systems. Regarding the composition of an encapsulation system, the encapsulated active agents can have a hydrophilic or lipophilic nature, using either materials derived from natural or synthetic sources. Several techniques have been proposed in the literature for encapsulation processes; nevertheless, there is not a specific method that could be regarded as a standard suitable for the different active agents and encapsulated systems (for Review, see Vieira et al. [34]).

Food safety must also be carefully considered. Algae can either produce (biogenic) or absorb (non-biogenic) and accumulate toxins from the environment. Biogenic toxins include nucleic acids, which are part of all living organisms. Algae contain 4-6% nucleic acid, which is a source of purines. These, if ingested by humans in excessive quantities, lead to an increase in the concentration of uric acid and, consequently, to a greater risk of contracting gout. To avoid this risk, the Protein Advisory Groups set the limit for intake of nucleic acids from unconventional sources at 2 g per day. Non-biogenic toxins, on the other hand, include heavy metals, which are a major problem in the large-scale production of microalgae. The levels of heavy metal accumulation are not fixed, but vary depending on the culture medium, possible contamination during the process, or even because of unsuitable analytical techniques [35].

An aspect of utmost importance when considering microalgae nutritional and functional features, is the complexity of the polysaccharide cell wall, which can limit the action of digestive enzymes, hindering the bio-accessibility or bioavailability of bioactive components. Since the wave of research excitement in microalgae, potential often relies on the protein fraction, and studies in protein digestibility, which can help estimate their availability, are needed as few authors have examined this aspect. For instance, Niccolai et al. [36] reported that digestibility varies greatly among species, with *A. platensis*, *Chlorella vulgaris* and *Chlorella sorokiniana* showing the highest values, while *Tetraselmis suecica*, *Phaeodactylum tricornutum*, and *Porphyridium purpureum* are the least digestible, which suggests that pre-treatments to disrupt the cell wall are often necessary. Nevertheless, the production process of microalgae already entails various energy intensive processes, which if operated through solar-power electricity, could reduce the energy footprint, and improve environmental performances [37]. Consequentially, all the treatments following the production should be as sustainable as possible and should consider microalgae bioactive potential, all factors that will drive the growth of microalgae use as ingredient for human consumption.

**Table 1:** Main functional effects ascribed to microalgae’s principal components.

	Functional Effect	References
**Proteins**		
**Bioactive peptides**	Hormonal activity, antioxidant, anticoagulant, antihypertensive, immunomodulatory, antimicrobial and cholesterol lowering functions	[30]
**Phycobiliproteins**	Anticancer, anti-inflammatory anti-oxidative, anti-viral, hepato-protective, neuroprotective effect	[38,39]
**Pigments**		
**Carotenoids**	Anti-inflammatory, anticancer activities, antioxidant effect	[32]
**Chlorophyll**	Accelerate wound healing antioxidant properties	[40]
**Fucoxanthin**	Antioxidant, Antidiabetic, Anti-Inflammatory, and Anti-Obesity activities	[41]
**Astaxanthin**	Antioxidant and antimicrobial activities	[42]
**Phycocyanin**	Antioxidant, anticancer, anticarcinogenic, anti-inflammatory, neuroprotective, hepatoprotective, immunomodulatory, and reno-protective pharmacological effects, and antidiabetic potential	[43]
**Lipids**		
**Eicosapentaenoic acid and docosahexaenoic acid**	Reduction of complications in cardiovascular, arthritis, and hypertension; hypolipidemic activity	[31]
**Sterols**		
**Phytosterol**	Immunomodulatory, anti-inflammatory, anti-hypercholesterolemic, antioxidant, anticancer and antidiabetic effects	[44]
**Polysaccharides**	Immunomodulatory, antioxidant, anti-inflammatory, anti-tumor, prebiotic, and antimicrobial activities	[38,45,46]
**Vitamins**	Antioxidant activity	[47]
**Phenolic compounds**		
**Apigenin**	Autophagy induction in leukemia cells	[48]
**p-coumaric acid**	Antioxidant activity	[49]

### 3.3. Metabolites Extraction

Although showing several beneficial effects, the use of algal biomass in its entirety brings with it some limitations: unpleasant odors and a green color, which change the traditional features of food.

To avoid these issues, industry has focused its attention on microalgae metabolites. Various high-value compounds such as carotenoids, β-carotene, astaxanthin, cantaxanthin, lutein, and fatty acids can be extracted from the biomass and used in nutrition. The first step for the extraction of the metabolites entails the cell wall breaking, and since their composition varies from species to species, there is no single technique to break them down. Nevertheless, mechanical, or non-mechanical methods can be used. Mechanical methods include the use of autoclaves, spray-drying, bead mills, cell homogenizers and ultrasound, whereas the use of acids, bases, enzymes, organic solvents, freezing, and osmotic shock are some of the non-mechanical methods [22]. For the extraction of metabolites for food use, ethanol 96%, hexane, or a hexane-ethanol 96% mixture are used [22]. However, the removal of the solvents from the biomass is an important aspect to consider in avoiding toxicity phenomena [35].

Cuellar-Bermúdez et al. [50] studied the ability of three solvents (ethanol, hexane, and acetone) to remove microalgae odors without altering nutritional properties. The extraction with ethanol was the least effective in eliminating unpleasant odors, followed by acetone and hexane. The extracts were then separated by chromatography to identify the compounds responsible for the fishy odor, which were identified as 1.4:3.6 anhydro-α-d-glucopyranose, palmitic acid methyl ester and hexadecanamide. None of the three solvents modified the nutritional profile of *A. platensis*: only an increase in tryptophan concentration was detected in the ethanol-treated sample, compared to the control [50].

Among the metabolites extracted from microalgae, astaxanthin, which is considered GRAS by the FDA, is currently widely commercialized for food applications. This carotenoid can be produced by *Haematococcus pluvialis* under stress conditions in closed culture systems with a 5–7 day “reddening” cycle, conducted in open culture ponds [51]. Alternatively, astaxanthin can be produced in a mixotrophic-based indoor system [52].

The European Commission has recently authorized the commercialization of food containing docosahexaenoic and eicosapentaenoic acids extracted from the microalgae *Schizochytrium* sp. These fatty acids are the two most important alpha linolenic acid derivatives for the human body. Additionally, it has been proved that polyunsaturated fatty acids play a role in preventing cardiovascular disease, inflammation, autoimmune diseases, depression, and neurological diseases [53].

Phenolic compounds are also considered important microalgae derived compounds used as food ingredients, since they can combat free radicals, which are harmful to the human body [54]. Phenolic compounds are extracted using organic solvents or subcritical-water extraction [55], yet a new extraction technique based on a combination of solid-phase/supercritical-fluid for Spirulina PCs was studied by Klejdus et al. [56].

Subcritical-water extraction can also be coupled with ultrasound to extract peptides (180–5000 Da) [57]. Many bibliographic studies can be found on PC proprieties in Spirulina and Chlorella. One of the earliest research projects concerned the use of Spirulina maxima for the preparation of pharmaceutical products to accelerate wound healing, yet inhibitory effect on oral carcinogenesis in animal models, and the ability to act indirectly on total and high-density lipoprotein cholesterol values and to improve blood serology parameters and IQ scores were also described [58,59].

The ethanolic fraction, extracted from *Chlorella pyrenoidosa* using 55% absolute ethanol at a 1:10 ratio (*w*/*v*), showed anti-hyperglycemic activity in male rats fed a high fat diet, as assessed by oral glucose loading test [60]. Chlorella is also a source of peptides with free radical scavenging properties like Val-Glu-Cys-Tyr-Gly-Pro-Asn-Arg-Pro-Gln-Phe from *C. vulgaris* [61] and Leu-Asn-Gly-Asp-Val-Trp from *Chlorella ellipsiodea* [62].

Metabolites extraction can also be the result of enzymatic treatments. Verdasco-Martín et al. [63] studied the effect of four different enzymes on Spirulina biomass: two proteases (Alcalase^®^ and Flavourzyme^®^) for the degradation of membrane proteins and lipoproteins, and two endo- and exo-glucanases (Ultraflo^®^ and Vinoflow^®^) to breakdown the sugar polymer structure peptidoglucan. Among the enzymes, Alcalase^®^ gave the highest extraction yield of hydrophilic compounds, because of its effective degradation of membrane proteins, lipoproteins, and peptidoglycan. Both the extract composition and the amount of extracted biocomponents depended on the temperature, enzyme charge and type, pH, and duration of enzymatic pre-treatment of the biomass [63]. Alcalase^®^ significantly increased the recovery percentage of amino acids [63], but also proved to be the best in releasing protein hydrolysates of *A. platensis*, with the highest antioxidant activity [64,65]. The antioxidant peptide VTAGLVGGGAGK exhibited, in fact, the highest ABTS (2,2′-azino-di-[3-ethylbenzthiazoline sulfonate]) radical scavenging activity, hydroxyl radical scavenging activity and ferrous ion chelation activity with EC50 value of 1.08 mg mL^−1^, 1.35 mg mL^−1^ and 1.24 mg mL^−1^, respectively. A slight reduction in antioxidant activity was observed when bioprocessed *A. platensis* was subjected to gastro-intestinal digestion (GID). Indeed, to exert biological effects in vivo, bioactive peptides must undergo a GID process, which will significantly impact the bioactivity of peptides, and since ethics regulations on animal studies are strict and with high costs, the in vitro simulated GID model has become a rapid and well-accepted approach to mimic the digestion process of peptides prior to conducting in vivo studies. Conversely, the ACE (Angiotensin I-Converting Enzyme) inhibitory activity of peptides from *A. platensis* proteins (hydrolyzed with trypsin) was not affected by pH, heat treatments and GID [65].

Enzymatic hydrolysis was combined with a bead milling process by Alavijeh et al. [66] to fractionate the major valuable biomass components of *C. vulgaris*. It was observed that, on applying enzymatic treatments on bead milled biomass, the recovery yield of proteins, carbohydrates, and lipids improved significantly. In particular, the best results were obtained with the use of the lipase at 37 °C and pH 7.4 for 24 h, releasing 88% lipids in the solid phase, while 74% carbohydrate and 68% protein were separated in the liquid phase.

Hydrolysis of *A. platensis* phycobiliproteins with trypsin, on the other hand, produces fragments capable of inhibiting dipeptidyl peptidase-IV (DPP-IV), a serine hexopeptidase, considered a promising target for type 2 diabetes mellitus treatment [67]. DPP-IV is also inhibited by the peptide Leu-Arg-Ser-Glu-Leu-Ala-Ala-Trp-Ser-Arg, for which a beneficial action against hyperglycemia has also been observed, as it inhibits α-amylase and α-glucosidase [68].

### 3.4. Bioprocessed Microalgae

The use of the whole microalgae biomass can be achieved with different strategies aiming at avoiding issues related to flavor and odor. Several studies have indeed investigated the possibility of removing typical microalgae odors without altering their nutritional properties.

Fermentation with *Bacillus subtilis* and *Lactiplantibacillus plantarum* also showed a significant removal of the volatile compounds of Spirulina, while acetoin was generated as the dominant volatile compound responsible for the creamy fragrance of the fermentation products, along with other volatiles, such as ethyl L(-)-lactate, lactic acid and (R,R)-2.3-butanediol [69]. A more recent study successfully investigated the possibility of reducing pyrazine, ketone, and aldehyde concentrations in Spirulina powder through fermentation with yeast species including *Debaryomyces hansenii*, *Kluyveromyces marxianus*, and *Saccharomyces cerevisiae* [70]. Indeed, profiles characterized by lower content of those compounds responsible for “seaweed” and “umami” flavor and higher content of those responsible for “fermented” attributes usually distinguish fermented microalgae [69,70,71].

Lactic acid fermentation can also be used to improve the antioxidant activity of *A. platensis*. An increase of DPPH (2,2-diphenyl-1-picrylhydrazyl) radical scavenging activity (up to almost 80%) was observed after fermentation with a probiotic strain of *L. plantarum* ATCC8014, probably due to the increase of phenolic compounds concentration (from 4.5 to 18.9 mg g^−1^ gallic acid equivalents after 48h of fermentation) [72]. Increases of the radical scavenging activity on DPPH and ABTS were also observed when *A. platensis* was fermented with *L. plantarum* T0A10 and subjected to enzymatic hydrolysis with Alcalase^®^ [29]. In this study, freeze-dried Spirulina biomass bioprocessed with Alcalase^®^ and *L. plantarum* T0A10 also showed promising antimicrobial activity against fungal spoilage *Penicillium roqueforti* and food pathogen *Escherichia coli*. Moreover, higher protein availability and digestibility, by means of the increase of polypeptides and free amino acid concentrations, have been achieved when fermentation was used alone [69] or combined with enzymatic treatment [29]. Although, compared to seaweeds, fermentation of microalgae is much less studied, the modulation of the antioxidant properties through fermentation either by yeasts [73] or lactic acid bacteria [74,75,76], is the most commonly evaluated aspect. Indeed, increases of radical scavenging activity, oxygen radical absorbance capacity, and ferric reducing antioxidant power or inhibition of lipid peroxidation in vitro are the most common results obtained. Nevertheless, since in vitro assays can only be considered as predictive tools of the functional effect which can be obtained in vivo, methods assessing fermented microalgae antioxidant properties on cellular models have been evaluated. Indeed, fermentation of *S. maxima* with *L. plantarum* HY-08, combined with ultrasonic extraction at 40 kHz, led to neuroprotective activity in mice, preventing memory impairment caused by oxidative stress [77]. Such results were ascribed to the synergistic effects of the high amounts of β-carotene and other biologically active substances in the extract released during fermentation. Similarly, fermentation with *Levilactobacillus brevis* BJ20 of a sterilized mixture of the microalga *Pavlova lutheri* and the yeast *Hansenula polymorpha*, allowed a product with an extremely high antioxidant potential, also showing no cytotoxicity in mouse macrophages (RAW264.7 cell), human myeloid cells (HL60), and human fetal lung fibroblast cell line (MRC-5) [78]. Besides, when the same microalga was fermented with *H. polymorpha*, a bioactive peptide exhibiting anti-osteoporosis activity was also released [79].

The effects of supplementation with Chlorella, fermented with baker’s yeast and lactic acid bacteria in growing pigs was also studied [80]. Results indicated that the inclusion of fermented Chlorella in the diet could improve the growth performance, nutrient digestibility, fecal microbial shedding (lower *E. coli* and higher *Lactobacillus* spp.) and decrease gas emission in growing pigs when compared with the group fed the basal diet [80].

**Figure 2:**
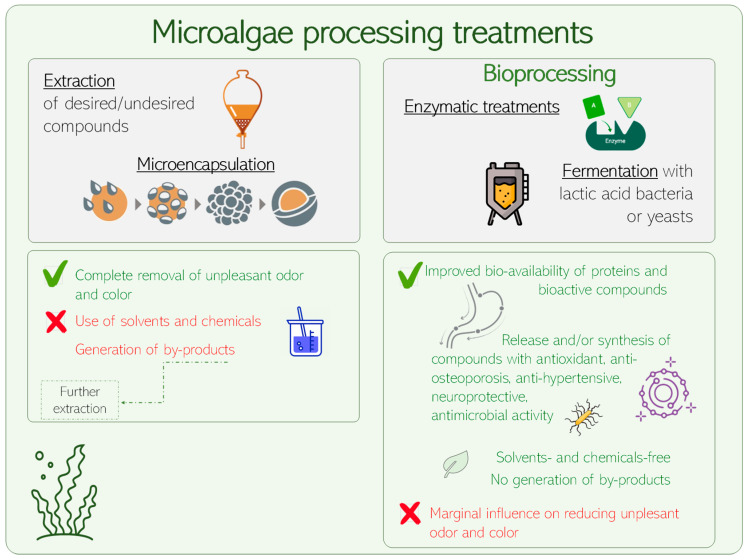
Illustration of the main advantages and limitations of microalgae processing treatments commonly proposed to improve their nutritional and functional potential.

Beside the selection of algae strains with low content or absence of pigments (i.e., *Prototheca* genus), and since the typical green color of algae is a limitation for the use of the whole algal biomass, hardly solved with bioprocessing treatments, X-rays or UV, chemical or physical agents (heat treatment) can be used. Nevertheless, none of these techniques guarantee the maintenance of the quality, richness and/or diversity of other components of interest, which is why, when choosing the right processing treatment to apply, a conscious decision, considering both the advantages and limitations of said treatments should be considered (Figure 2).

### 3.5. Unprocessed Microalgae as Prebiotic

*A. platensis*, when used as it is, can act as a prebiotic for lactobacilli in food matrices by preserving their availability [81] and stimulating their growth [71,82]. Indeed, it has been recently reported that sterilized algae stimulated the growth of *Lacticaseibacillus casei* and *Lacticaseibacillus rhamnosus* [83]. The presence of *A. platensis* also improved the formation of those compounds responsible for aldehydic/ethereal, buttery/waxy, alkane and of course fermented aromatic notes. *Lact. casei* 2240 produced, in sterilized Spirulina, a high amount of diacetyl and acetoin and *Lc. rhamnosus* GG affected the content of these compounds more prominently. In addition, buttery/waxy and fermented aromatic notes were also influenced by stabilization treatment [83]. The authors also studied the ability of *A. platensis* to enrich culture media and promote the development of acidifying strains. For this purpose, sterilized *A. platensis*, rehydrated in water, was added at 0.25% and 0.50% to three different media: tryptic soy broth, reconstituted skim milk, and commercial soy drink. Then, the growth of *Lc. rhamnosus*, *S. thermophilus*, and *Lactococcus* sp. was monitored. The biomass proved to be a suitable matrix for fermentation, showing a bacterial growth of more than 2 log CFU g^−1^ in 48 h [83].

## 4. Microalgae as Ingredient in Food Making

The number of microalgae-containing foods available on the market is steadily increasing. Indeed, between 2015 and 2019 approximately 13,090 new food and beverages containing algal ingredients were launched in the global and European (5720) market. These new products included 79% in foods and 21% in beverages [7]. In Asia it is possible to buy crackers filled with Spirulina and in America an organic cucumber and avocado soup containing Spirulina, whereas a protein bar with Spirulina is commercialized in Canada. British consumers can also have breadsticks containing 2% Spirulina [20]. Nevertheless, most of the edible microalgae currently commercialized are sold as dietary supplements, as dried powder, capsules, or flakes. The very low concentrations used in some products suggest that microalgal biomass is used as a coloring agent or for marketing purposes, rather than for potential nutritional advantages [20]. Although it will not be a topic of pertinence for this review, microalgae potential to be used as ingredient for meat analog has been explored. Still, chlorophyll removal, cell disruption, protein extraction and purification, and a series of treatments to mimic both meat texture and flavor, are necessary, rendering the feasibility and, most of all, the sustainability of the whole process quite questionable (for review, see Fu et al. [84]).

On the contrary, the fortification of cereal-based foods with unconventional matrices is a topic widely investigated by the scientific community in recent decades [85]. Several studies showed the potential benefits of adding microalgae in foods, due to their high biological and nutritional value. However, if on the one hand, supplementation of fruit, vegetables, legumes, grains, food by-products or their extracts, raw or processed, proved to be a good strategy to enhance the content and bioavailability of the nutrients, bioactive compounds, and dietary fiber of cereal foods, on the other hand, this entails technological issues [85]. As for such matrices, the incorporation of microalgae in foods implicates both advantages and challenges (Table 2).

Currently there is no agreement upon the maximum percentage of Spirulina addition. Although it is highly unlikely to establish the amount of microalgae to be added to food so that sensory and technological properties are not hindered, since this is strictly dependent on the type of food and recipe formulation, it would be appropriate to establish evident criteria based on microalgae composition, which could allow complementary addition (i.e., balanced amino acid composition in cereal- or legume-based products where some amino acids are limited).

### 4.1. Bread and Leavened Products

Microalgae have been generally introduced into bread formulations to increase the consumption of non-animal proteins in the human diet. Indeed, the addition of Spirulina at percentages between 1 and 5% led to an amelioration of the nutritional balance of common [86] or gluten-free [87] breads, especially at the highest percentages. The nutritional improvement is mainly ascribed to the protein fraction, both in terms of quantity (up to 39% more than control bread) and quality, with increases in essential amino acids like threonine, methionine, isoleucine, and leucine [87]. The level of fortification was also increased up to 10% aiming at exploiting the valuable metabolites included in Spirulina platensis [108,109]. Besides the expected increase in the protein content (up to 11.63%) and in-vitro digestibility [109], the incorporation of Spirulina led to a more complex profile of volatile aromatic compounds (up to 13 new detected) and positively affected the mineral presence (calcium, magnesium, and iron) as well as the inhibition of mold growth, while being accepted by a panel test [108,109]. Similarly, the fortification of gluten-free bread with Spirulina led to a product with more abundant medium organic acids and exclusive bioactive compounds (thymol, borneol, and nicotinic acid), which were correlated with the prebiotic activity of spirulina breads [110]. Notwithstanding, it was observed that microalgae incorporation decreases bread volume due to the reduction of the hydrated starch. Indeed, the algal biomass could compete with the starch for water. A recent study highlighted that Spirulina concentrations higher than 2% may limit the workability of the dough due to excessive stickiness [111]. Nevertheless, a fortification up to 2% (*w*/*w*) reduces the gluten content without compromising the rheological parameters of the dough [111]. Although higher microalgae concentrations often result in negative effects on dough rheology, as well as on bread texture and flavor, no impact on yeast fermentation nor on the time required for fermentation seems to be induced by the biomass addition [88].

Among algae, Spirulina is the most commonly utilized for inclusion in bread, whereas only a limited number of studies have evaluated different algae species. For example, Graça et al. [88] recently studied the consequence of using *C. vulgaris* from 1 to 5% in wheat flour dough to study its rheology and bread textural properties. It was observed that incorporation of the microalgae at concentrations up to 3% produced a positive impact, strengthening the gluten network. The incorporation of Chlorella in wheat flour seemed to contribute to more extensible dough (L), favoring the action of gliadin. In contrast, it had an adverse effect on the glutenin action, decreasing the dough tenacity (P), at over 3 g/100 g. Blends with over 3 g/100 g showed a reduction of P/L ratio, which provides information about the elastic resistance and extensibility balance of wheat flour dough. The authors concluded that these mixtures are not suitable for bread making, but they can be used for unleavened products, such as cookies and biscuits. On the contrary, when used in gluten-free bread, *Nannochloropsis gaditana* L2 and *Chlamydomonas* sp. EL5 were found to be responsible for a structuring effect on the bread texture, mainly due to the high protein content. The technological advantages were also accompanied by nutritional improvements (protein, lipids, and iron contents) [112].

The influence of microalgae incorporation in other products was also investigated. “Crostini” were enriched with 2%, 6% and 10% (*w*/*w*) A. platensis F&M-C256 leading to products with higher nutritional value (protein and phenolics content and antioxidant activity) [89]. Nevertheless, a significantly lower value of in vitro dry matter and protein digestibility between A. platensis F&M-C256 “crostini” and the control was found, while the overall acceptability decreased with increasing A. platensis F&M-C256 addition.

### 4.2. Other Baked Goods

Although still debated, studies on bread fortified with microalgae suggest that changes in the rheological and technological properties of the products might make this ingredient more suitable in unleavened products where friability, over elasticity, is the main textural attribute required. Indeed, it was found that A. platensis provided a significant structuring effect in cookies, so that hardness was significantly reduced after eight weeks of storage [90]. Spirulina was also used, with different levels of inclusion (0, 1, 3, and 6%) to produce shortbread biscuits with the aim of assessing their physicochemical properties and consumer acceptability during storage [113]. Besides the nutritional and functional improvements already discussed for leavened products, the fortification led to significantly harder and darker biscuits (compared to the control) during the whole period of storage, but still acceptable to consumers [113].

Nevertheless, from the research on bread enriched with Spirulina, the obvious and sometimes unpleasant contribution of microalgae emerged on the product color, proportional to addition [86,87]. For this reason, among the aspects deepened, the impact of microalgae on baked goods’ color was studied by evaluating different microalgae species [89] or recipe formulations [114]. Cookies fortified with *S. platensis*, *C. vulgaris*, *T. suecica*, and *P. tricornutum* at concentrations of 2 and 6% showed different green tonalities, depending on the microalga used, varying from a blueish-green (*A. platensis*) to a brownish-green (*P. tricornutum*) [90]. Overall, the color stability of foods containing microalgae seems to be very high, as also demonstrated by other authors [91,94].

When *A. platensis* was used to improve the nutritional profile of gluten-free cassava doughnuts [109], inverted sugar was used to mask the green color linked with microalgae addition, by increasing the rate of the Maillard’s reaction. The overall nutritional quality of the doughnuts was improved by the fortification (>5%) with Spirulina, ameliorating protein, mineral, fiber, and lipid composition. The substitution of sucrose with inverted sugar also promoted a decrease in the shearing force, related to the favored interactions between proteins and water in the dough [109]. Besides raising the amount of protein, compared to their respective controls, significantly higher total phenolic content and in vitro antioxidant capacity in microalgae-based cookies were found [87,90]. Similar results were reported by Hossain et al. [93] after incorporating *H. pluvialis* into cookies to modulate glycemic response and enhance nutritional aspects. Indeed, results showed a reduction in the rate of glucose released during in vitro digestion for cookies with 15% astaxanthin, compared to control. Other microalgae have also been included in baked goods. For instance, *Nannochloropsis* was recently used to produce functional crackers [115] without affecting the physicochemical properties of the end products expected by a darker and greener color. A higher number of bio-accessible polyphenols and in vitro antioxidant capacity was also achieved while showing a competitive sensory profile [115].

### 4.3. Beverages and Soups

In the past few years, microalgae have been proposed as ingredients in sweet and savory beverages and soups. Beyond the nutritional improvement, the addition of *Spirulina* sp. at a concentration of 750 mg/100 g to a chocolate flavor shake-type powdered food did not considerably affect the sensory properties of the product, thus not hindering its acceptability, but causing a decrease in shelf-life [95]. Microalgae-containing plant-based soups have also been formulated. For example, Castillejo et al. [96] analyzed smoothies prepared with grapes, broccoli, cucumber, and supplemented (at a concentration of 2.2%) with several edible algae, including *C. vulgaris* and *Spirulina* sp. After seven days, all tested smoothies supplied 50–60% of the recommended intake of vitamin C. Chlorella and Spirulina smoothies showed the highest B12 vitamin content (33.3 and 15.3 μg/kg, respectively), which remained constant during shelf-life; however, due to the stronger marine odor and flavor of *C. vulgaris* smoothies compared to those containing Spirulina sp., a lower overall quality score was observed. Low acceptability scores, especially at high concentrations, were also observed for a broccoli soup enriched with *Spirulina* sp., *Chlorella* sp., or *Tetraselmis* sp. [97]. Nevertheless, microalgae incorporation determined an increased content of polyphenols and higher antioxidant capacity resulting in a higher content of bio-accessible polyphenols, calculated after a simulated gastrointestinal digestion. Increased in vitro and ex-vivo antioxidant activity was also observed in a vegetal soybean drink or in water enriched with *A. platensis* biomass, then fermented with *L. plantarum* LAB8014 [98]. Compared to control drinks, the addition of *A. platensis* biomass to the beverages determined an average reduction of 25.5% in intracellular oxidation level in Saccharomyces cerevisiae cells, further reduced (36.9%) by fermentation [98]. Spirulina was also used in combination with other ingredients in fruit-based juice. Indeed, a juice based on cantaloupe and pear was fortified with wheat germ powder and spirulina (1%) with little effect on pH, acidity, and formalin index, yet brix, dry matter, and protein content were affected. The addition of spirulina and wheat germ powder also changed the amounts of antioxidant capacity (from 90 to 98%), total phenol (from 4 to 22 mg GAE/g), and flavonoid content (from 5 to 15 mg/L) in the functional beverage. Moreover, spirulina did not affect the rheological properties [116].

Due to its nutritional profile, spirulina can also be added to sports drinks affecting total sugar, ascorbic acid and antioxidant activity of the product during storage under refrigerated conditions [117]. Indeed, the addition of spirulina extract to sports drinks helped improve the product’s nutritional value because of its high ascorbic acid concentration and improved antioxidant activity [117].

### 4.4. Snacks

As for other food products, increases in proteins, lipids, and minerals content after incorporation of Spirulina biomass in snacks were observed by Lucas et al. [99] and Tańska et al. [100], though a clear understanding on how Spirulina affects physical parameters, such as expansion index and hardness, or sensory properties is hard to identify, since the former did not present significant differences compared to the control, whereas the latter did. Indeed, the snacks produced by Lucas et al. [95] had a high acceptability index (82%) and physical and microbiological stability during 12 months of storage, whereas for those of Tańska et al. [100], the sensory score was lower than the control and decreased with the increase of added microalgae. It is, however, likely that the different formulations (rice and corn flour in one case and just corn in the other), even with an equal percentage of Spirulina (around 2%), might have interacted differently with the microalgae.

Nevertheless, since the extrusion process entails the exposure to heat and high pressures and a loss of nutritional quality (e.g., loss of thermolabile vitamins, denaturation of proteins or oxidation of phenolic compounds), some authors evaluated the possibility of using *S. platensis* as dragée before inclusion in corn snacks, which allowed a product with a more balanced nutritional profile, appreciated by panelists [118]. The dragging process determined a decrease in the snack energy value, while increasing vitamins, protein, minerals, essential and non-essential amino acids, fatty acids, as well as flavonoids and total anthocyanin content. Along with polyphenols, peptides may also display antioxidant features and one of the ways to generate bioactive peptides in microalgae, as already covered above, is subjecting them to enzymatic hydrolysis. Indeed, da Silva et al. [119] prepared a Spirulina hydrolysate using Protemax, a se,- from *Bacillus lichenformis*. Peptide fractions having MW lower than 4 kDa were then used in the formulation of extruded snacks, enhancing the ABTS+ inhibition (7.45 μmol TEAC/g) and increasing the reducing power (12.09 μmol Trolox/g) compared to the untreated-Spirulina snacks, thus reiterating the potential of bioprocessing technologies.

Examples of snacks produced with microalgae other than Spirulina were provided by Batista et al. [101], who studied the effects of microalgae-fortification in artisanal wheat crackers. These snacks were enriched with *A. platensis*, *C. vulgaris*, *T. suecica*, and *Phaeodactylum tricornutum* at two incorporation levels (2 and 6%). The 2% formulation with *A. platensis* and *C. vulgaris* presented high digestibility and high sensory analysis scores, whereas 6% formulation presented a significantly higher protein content, so they could be claimed as a “source of protein” according to the Regulation (EC) No. 1924/2006. *T. suecica* and *P. tricornutum* crackers showed high phenolic content and antioxidant activity but low sensory scores; nevertheless, extensive toxicological data are required to approve *P. tricornutum* utilization as a novel food [101].

### 4.5. Pasta

As for leavened bakery products, the strength of the gluten network is one of the main factors to be considered during the production process of pasta, since it affects protein loss in water during pasta cooking, and the ability to withstand cooking is a feature highly appreciated by consumers. For this reason, fortifying pasta with non-gluten containing matrices often leads to a deterioration in the structure; however, when *C. vulgaris* and *S. maxima* were used for spaghetti, changes in the protein network were found to determine a better quality [99]. On the contrary, De Marco et al. [102] found opposite results. Indeed, compared to semolina pasta, the weaker structure of fortified pasta leached more solids into the cooking water, increasing cooking residues and decreasing the optimal cooking time. Once again, such a difference is most likely due to a different percentage of addition, up to 10-fold higher in the latter research. Indeed, as confirmed by Raczyk et al. [120], a decrease in textural and sensory attributes for pasta fortified with Spirulina is proportional to the percentage of semolina replacement. Nevertheless, since the greatest nutritional improvements are achieved at the highest percentages, future research should face this issue, especially in Western Countries. As a matter of fact, the human component in sensory analysis has a relevant weight that cannot be overlooked, since researchers from India found opposite results. When they used Spirulina at incorporation levels up to 15% in semolina pasta, acceptability scores remained high despite texture parameters and cooking quality decreasing, compared to control pasta [121].

Apart from technological and organoleptic issues, a modification of the protein profile might also hinder the nutritional quality of pasta (e.g., its protein digestibility). The digestibility of algal proteins is influenced by the presence of anti-nutritional factors, either phenolic molecules or polysaccharides, which can bind proteins forming insoluble compounds that inhibit the activity of proteolytic enzymes [102]. Otherwise, different biochemical composition of the microalgal biomasses, as well as the desiccation treatments they are subjected to, might also affect protein digestibility.

Indeed, when Fradinho et al. [122] used *A. platensis* in the production of gluten-free fresh pasta, in vitro protein digestibility showed different trends based on the biomass used (F&M-C256 and Ox Nature, resulting from different drying procedures). Pasta supplemented with A. platensis biomass from Ox Nature (commercial spray-dried biomass) presented higher protein digestibility than control pasta, while pasta with *A. platensis* F&M-C256 (centrifuged and lyophilized biomass) at 3% incorporation level slightly reduced the protein digestibility. The authors explained the different protein digestibility to the different biochemical composition of the microalgal biomasses resulting from different culture conditions adopted during cultivation [122].

Reports of enhancement of the nutritional quality of semolina or gluten-free pasta enriched with *A.* platensis can also be found in the literature, mainly ascribed to an increase in the phenolic content and related antioxidant activity compared to control pastas [102,104]. In addition to the enrichment with phenolic compounds, when microalgae are added to pasta, a modification of the fatty acid profile occurs. Indeed, *Nannochloropsis* sp. biomass was used to fortify pasta with polyunsaturated fatty acids, so that the consumption of the 40%-enriched pasta represented roughly the minimum daily intake recommended for eicosapentaenoic acid and docosahexaenoic acid, remaining stable even after the drying and cooking process [123].

### 4.6. Dairy Products and Analogous

The effect of the addition of microalgae at different concentrations in dairy products and analogous (e.g., yogurt, ice-cream, cheeses, and vegetable substitutes) was studied by several authors and recent reviews focused on the application of microalgae to produce novel dairy products; below are reported those examples more of pertinence to this review which were not covered by Hernández et al. [107] and Garofalo et al. [124].

The literature agrees on the ability of *C. vulgaris* and *A. platensis* to increase the viability of probiotic strains of *Lactobacillus acidophilus* LA-5 and *Bifidobacterium lactis* BB-12 [106] and lactic acid bacteria such as *Lev. brevis* ŁOCK 0944 [125], as well as many others [126]. It was indeed hypothesized that the presence of extracellular products (protein, peptides, amino acids, minerals, vitamins, etc.) brought about by the microalgae promotes microbial growth, thus increasing the synthesis of organic acids and overall acidification [125,126]. However, since high concentrations of microalgae confer an unpleasant color and graininess, supplementing fermented milk with flavorings succeed. Indeed, the addition of 10% of papaya to a yogurt containing 6% of *Arthrospira* allowed a higher sensory score [127]. Alternatively, to avoid these drawbacks, compounds extracted from microalgae could be used. For example, the effect of phycocyanin pigment on the physicochemical and microbial properties of fermented milk during 21 days of storage was recently studied by Mohammadi-Gouraji et al. [128], showing that phycocyanin pigment improves yogurt texture, increasing firmness and color stability, with no negative effects on yogurt starter cultures.

Instead of using flavorings, coloring, and texture improvers, limiting both unpleasant odors and colors can be achieved with microencapsulation of microalgae. Indeed, da Silva et al. [105] studied the incorporation of *S. platensis* in yogurts in three forms: free, microencapsulated in maltodextrin (SM), and microencapsulated in maltodextrin crosslinked with citric acid (SMA). Although the free form of *S. platensis* showed the highest DPPH scavenging activity and reducing power when compared to microspheres, SMA showed better antioxidant and anti-inflammatory activity than SM. None of the assayed samples showed cytotoxicity, which made them suitable for food applications. On the contrary, encapsulation of *S. platensis* was found to increase the antimicrobial activity against *Ps. aeruginosa*. Yogurts prepared with encapsulated *S. platensis* presented a more homogeneous appearance, with attenuated green color, and without the unpleasant fish-like odor [105].

Fermentation might also be used to increase the functionality of yogurt-like beverages. Fermentation of *A. platensis* with a pool of lactic acid bacteria (*Lactobacillus acidophilus*, *Bifidobacterium bifidum*, *Lact. casei*, *Bifidobacterium infantis*, *Bifidobacterium longum*, *Lac. lactis*) was also explored to achieve protective effects on human keratinocytes (HaCa T cells) subjected to UVB-induced oxidative stress [129]. Even though this study showed that microalgae fermentation has applications in suntan lotion and skin-care products for preventing ultraviolet radiation or damage due to urban smog, it could be hypothesized that their potential goes beyond the cosmetic industry and should be further explored within the food sector.

## 5. Conclusions and Future Perspectives

Currently, the consumption of microalgae is mainly represented in dietary supplements, while fortified foods are slowly making their way into the market, with *A. platensis* and *C. vulgaris* being the most commonly employed. Even among supplements, the production process highly affects the overall quality of the microalgae, from protein quality and quantity to the oxidation of lipids and pigments, with repercussion on the final product and its price. Therefore, more thought should be put into considering the method to apply and, although some techniques entail expenses seldom accepted at industrial level, the cost-benefit ratio represents the needle of the scale.

The concept of fortified foods, however, stands behind the ability to meet nutritional recommendations and modulate the diet towards healthier choices while addressing sustainability concerns. Compared to conventional items, foods fortified with microalgae are mainly characterized by higher content in proteins, proportional to their addition, yet, on the other hand, the fortification often corresponds to a worsening of the technological and sensory properties of the products, especially at percentage of addition higher than 3%. Poor digestibility, reduction of volume, low acceptability, and reduced shelf-life are among the most common disadvantages as consequences of fortification. Nevertheless, several treatments have been exploited, from the extraction of the desired/undesired compounds to microencapsulation, passing through bioprocessing technologies such as enzymatic treatment features, and fermentation, each of them with their own pros and cons. Extraction and microencapsulation are probably the most appropriate techniques to avoid undesirable features, but entail the generation of by-products or the use of chemicals whose sustainability is doubtful and long-term unacceptable. Nevertheless, aiming at valorizing the extraction of by-products, the concept of biorefinery, which relies on the application of a sequential process of extraction techniques in which diverse compounds are effectively recovered, has emerged in recent years, and needs further investigation and application. Bioprocessing technologies, however, are not only known for their low environmental and physiological toxicity but can also be tailored to achieve more specific advantages in terms of nutritional and functional properties (e.g., synthesis or release of antioxidant, antimicrobial, anti-hypertensive compounds) while limiting the impact on sensory properties. Still, although the literature is full of examples of foods fortified with microalgae, and the application of bioprocessing technologies has proven to be helpful in mitigating their drawbacks, little can be found on the use of bioprocessed microalgae as ingredients, hence this topic should be further exploited, and new solutions sought.

Microalgae are excellent candidates to be a food of the future, but for this to happen, more effort should be put into finding the right bioprocessing treatment that can allow the use of the whole biomass and be cost-effective while bringing about features that go beyond the mere increase of proteins. Research on protein digestibility, which can help estimate availability, is needed and, so far, only a few authors have examined this aspect, focusing only on in vitro techniques. Hence it would be appropriate to implement the knowledge with data from ex vivo and in vivo studies on the subject. Moreover, further studies exploiting bioprocessing technologies, such as fermentation and enzymatic treatments, should be performed with the aim of limiting the downsides arising from biomass processing (i.e., low protein digestibility as consequence of the desiccation treatment performed), as well as synthesizing new bioactive compounds (e.g., phenolic compounds or bioactive peptides) that can play a key role in physiological functions. Moreover, thanks to the nutritional and functional value, mainly related to the digestible proteins, fiber, vitamins, beta-carotene, iron, and omega 3/6 fatty acids, microalgae might be optimal candidates to produce food and drinks destined for specific consumers niches. For instance, the sport and energy drinks industry may benefit from the use of such ingredients.

## Figures and Tables

**Table 2:** Main nutritional, sensory, and technological effects of microalgae fortification in foods.

Product	Microalgae Used	Nutritional Effect	Sensory and Technological Effect	References
Bread	*S. platensis*	Increase of proteins, amino acids, and ashes content	Volume decrease, increase in crumb hardness and colour modification	[86,87]
	*C. vulgaris*		Positive impact on viscoelastic characteristics, with strengthening of the gluten network	[88]
Crostini	*A. platensis*	Increase of phycocyanin, total phenolic content and radical scavenging activity on DPPH radical	Appropriate volume after fermentation	[89]
Cookies	*S. platensis*, *C. vulgaris*, *Tetraselmis suecica*, and *Phaeodactylum tricornutum*	High protein and total phenolic content and in vitro antioxidant capacity	Colour modification depending on the microalga, decrease of hardness	[90]
	Chlorophyll extracted from *Chlorella* sp.	Increase of ash content.	Increase in weight, thickness, and moisture content. Decrease in pasting viscosities. Dark colour and increased hardness	[91]
	*S. platensis*	Increase of protein, ash, fibre, total phenolic content, and antioxidant activity	Interference in elastic net, colour modification	[92]
	Astaxanthin from *H. pluvialis*	Reduction of glucose released during in vitro digestion, increase in the total phenolic content, and antioxidant capacity	Reduction of height and diameter gain, and weight loss	[93]
	*C. vulgaris*		Poor colour stability during storage, increased firmness	[94]
Shake-type powdered food	*Spirulina* sp.	Increase of protein content, reduction of carbohydrates and lipid content	Shelf-life reduction	[95]
Smoothies with grapes, broccoli, cucumber	Several ground dried powder of edible algae, including *C. vulgaris* and *Spirulina* spp	Supplied 50–60% of the recommended intake of vitamin C	Stronger marine odour and flavour	[96]
Broccoli soup	*Spirulina* sp., *Chlorella* sp., or *Tetraselmis* sp.	Increase of polyphenols content and higher antioxidant capacity	Low acceptability scores	[97]
Vegetal soybean drink	*A*. *platensis*	*Increase of* in vitro and in vivo antioxidant activity, reduction of intracellular oxidation level		[98]
Extruded snacks	Spirulina	Increase of protein, lipid, and minerals content	High acceptability index	[99]
	Spirulina	Increase of nutritional qualities, phycocyanin was destroyed.	Decrease in lightness and expansion indices, increase in softness, greenness, and yellowness. Low acceptability scores	[100]
Artisanal wheat crackers	*A. platensis*, *C. vulgaris*, *T. suecica*, and *Phaeodactylum tricornutum*	Higher digestibility, antioxidant activity and protein content	Darker colour, reduction of crackers’ width and thickness	[101]
Pasta	Spirulina	Higher antioxidant activity, protein, and phenolic compounds content. Lower protein digestibility	Structure little compromised compared to control	[102]
	*C. vulgaris* and *S. maxima*	Decrease in protein content after cooking	Stability of colour after cooking, increase in firmness. higher acceptance scores than the control pasta.	[103]
	*A. platensis* (spray-dried or lyophilized biomass)	Increase in antioxidant activity. In vitro protein digestibility showed opposite trends for pastas obtained with either microalgae biomass	Accentuated green tonality, low cooking loss, increase in swelling index	[104]
Fermented milk	Microencapsulated *S. platensis*	Anti-inflammatory activity Antimicrobial activity against *Pseudomonas aeruginosa*	Absence of unpleasant fish-like odor, homogeneous appearance	[105]
	*C. vulgaris* and *A. platensis*	Higher viability of probiotic	Unpleasant flavour, colour changes to greenish or bluish	[106]
Ice-cream	*A. platensis*	Higher protein and fat content	Lower melting time, shorter first drop times, higher overrun, lower viscosity	[107]
Cheese analogous	*C. vulgaris* or *A. platensis*	Higher protein content, higher antioxidant activity	Lower melting index, higher firmness values	
Cheese	*C. vulgaris* or *A. platensis*	Higher antioxidant activity	Granular texture, bitter aroma and taste	

## Data Availability

No new data were created or analyzed in this study. Data sharing is not applicable to this article.
